# Association of Cerebral Amyloidosis, Blood Pressure, and Neuronal Injury with Late-Life Onset Depression

**DOI:** 10.3389/fnagi.2016.00236

**Published:** 2016-10-13

**Authors:** Min Soo Byun, Young Min Choe, Bo Kyung Sohn, Dahyun Yi, Ji Young Han, Jinsick Park, Hyo Jung Choi, Hyewon Baek, Jun Ho Lee, Hyun Jung Kim, Yu Kyeong Kim, Eun Jin Yoon, Chul-Ho Sohn, Jong Inn Woo, Dong Young Lee

**Affiliations:** ^1^Institute of Human Behavioral Medicine, Medical Research Center, Seoul National UniversitySeoul, South Korea; ^2^Department of Neuropsychiatry, Ulsan University HospitalUlsan, South Korea; ^3^Department of Neuropsychiatry, Seoul Metropolitan Government-Seoul National University Boramae Medical CenterSeoul, South Korea; ^4^Department of Neuropsychiatry, Seoul National University HospitalSeoul, South Korea; ^5^Department of Biomedical Engineering, Hanyang UniversitySeoul, South Korea; ^6^Department of Neuropsychiatry, Kyunggi Provincial Hospital for the ElderlyYongin, South Korea; ^7^Department of Neuropsychiatry, Changsan Convalescent HospitalChangwon, South Korea; ^8^Department of Nuclear Medicine, Seoul Metropolitan Government-Seoul National University Boramae Medical CenterSeoul, South Korea; ^9^Department of Radiology, Seoul National University HospitalSeoul, South Korea; ^10^Department of Psychiatry, Seoul National University College of MedicineSeoul, South Korea

**Keywords:** late-life onset depression, Alzheimer's disease, cerebral amyloidosis, neuronal injury, blood pressure

## Abstract

Previous literature suggests that Alzheimer's disease (AD) process may contribute to late-life onset depression (LLOD). Therefore, we investigated the association of LLOD with cerebral amyloidosis and neuronal injury, the two key brain changes in AD, along with vascular risks. Twenty nine non-demented individuals who first experienced major depressive disorder (MDD) after age of 60 years were included as LLOD subjects, and 27 non-demented elderly individuals without lifetime experience of MDD were included as normal controls (NC). Comorbid mild cognitive impairment (MCI) was diagnosed in 48% of LLOD subjects and in 0% of NC. LLOD, irrespective of comorbid MCI diagnosis, was associated with prominent prefrontal cortical atrophy. Compared to NC, LLOD subjects with comorbid MCI (LLOD_MCI_) showed increased cerebral ^11^C-Pittsburg compound B (PiB) retention and plasma beta-amyloid 1–40 and 1–42 peptides, as measures of cerebral amyloidosis; and, such relationship was not observed in overall LLOD or LLOD without MCI (LLOD_woMCI_). LLOD subjects, particularly the LLOD_woMCI_, had higher systolic blood pressure (SBP) than NC. When analyzed in the same multiple logistic regression model that included prefrontal gray matter (GM) density, cerebral amyloidosis, and SBP as independent variables, only prefrontal GM density showed a significant independent association with LLOD regardless of MCI comorbidity status. Our findings suggest AD process might be related to LLOD via prefrontal neuronal injury in the MCI stage, whereas vascular processes—SBP elevation, in particular—are associated with LLOD via prefrontal neuronal injury even in cognitively intact or less impaired individuals.

## Introduction

Late-life onset depression (LLOD), most commonly defined as depression occurring for the first time at the age of 60 years or older, has distinct clinical characteristics such as high medical comorbidity compared to early-life onset depression (ELOD; Salloway et al., 1996; Potter and Steffens, 2007). In addition, since cerebrovascular changes and vascular risk factors (VRFs) are more commonly observed in LLOD than ELOD subjects, the “vascular depression” hypothesis has been proposed as a possible biological etiology of LLOD (Alexopoulos et al., 1997a,b; Krishnan et al., 1997).

However, previous studies suggested that neurodegenerative processes, Alzheimer's disease (AD) in particular, may contribute to LLOD, which might be an early prodromal symptom of AD (Brommelhoff et al., 2009; Panza et al., 2010). According to prior epidemiological studies, the prevalence rates of depressive symptoms increased within 3 years preceding the diagnosis of AD dementia and were significantly higher in subjects with mild cognitive impairment (MCI) than normal controls (NC; Berger et al., 1999; Lopez et al., 2003). Such findings indicate that AD-related pathological processes, such as beta-amyloid (Aβ) deposition and related neuronal injury, might contribute to the occurrence of LLOD by increasing brain vulnerability (Weisenbach and Kumar, 2014).

In line with epidemiological findings, some studies reported altered plasma levels of Aβ peptides or their ratio, considered potential biomarkers for increased risk of AD or MCI (Song et al., 2011), in elderly individuals with depression (Pomara et al., 2006; Qiu et al., 2007), and proposed the term “amyloid-associated depression” as a prodromal manifestation of AD (Sun et al., 2008). However, since plasma Aβ biomarkers are indirect measures for probing cerebral amyloid burden and direction of change was inconsistent among previous studies, direct quantification of Aβ deposition in *in vivo* human brain is required to accurately assess the state of cerebral amyloidosis in LLOD subjects. Recently, several studies compared cerebral amyloid burden between subjects with late-life depression and NC using amyloid positron emission tomography (PET) imaging with ^11^C-Pittsburg compound B (PiB) or ^18^F-Florbetapir tracer (Butters et al., 2008a; Madsen et al., 2012; Wu et al., 2014). However, all these studies, which showed conflicting results, defined depression as a “late-life depression” (Butters et al., 2008a) or “lifetime history of major depression in the elderly” (Madsen et al., 2012; Wu et al., 2014); these definitions incorporate not only LLOD but also ELOD that recurs or continues in the late-life period (Panza et al., 2010). To date, whether cerebral amyloidosis is associated with pure LLOD and plays a critical role in the development of LLOD remains unclear.

Based on magnetic resonance imaging (MRI) studies, LLOD is closely associated with structural brain changes such as regional cortical atrophy, a marker for neuronal injury (Bobinski et al., 2000; Frisoni et al., 2010; Hampel et al., 2010; Jack et al., 2010, 2011; Vemuri and Jack, 2010; Whitwell and Vemuri, 2011), especially in prefrontal regions (Kumar et al., 1998; Almeida et al., 2003). Although the vascular process has been suggested as their cause (Alexopoulos et al., 1997a), structural brain changes in LLOD may be due to the neuronal injury associated with the pathological process of AD, which is initiated by cerebral Aβ deposition (Jack et al., 2010; Sperling et al., 2011). Thus, to clearly elucidate the integrative amyloid-associated neurobiological process that underlies LLOD, neuronal injury process identified by MRI-measured atrophy, as well as cerebral amyloid burden, should also be examined simultaneously along with the vascular risks. Nevertheless, to date, no studies have investigated cerebral amyloid burden and regional cortical atrophy in LLOD individuals by integrating the perspectives of AD and the vascular process.

Elucidating the underlying pathophysiology of LLOD, particularly its association with neurodegenerative process such as cerebral amyloidosis and neuronal injury in non-demented elderly individuals will provide us insight on predicting the prognosis of and developing the novel therapeutic approaches for LLOD in clinical practice (Pomara and Sidtis, 2007; Mahgoub and Alexopoulos, 2016). In the present study, we aimed to investigate whether LLOD is associated with cerebral amyloidosis and regional neuronal injury, along with the vascular risks, in elderly individuals with no dementia. We first examined regional cortical atrophy using voxel-by-voxel analysis to identify regional neuronal injury related to LLOD. Next, we investigated the association of LLOD with cerebral amyloidosis, as measured by both cerebral Aβ deposition and plasma Aβ peptides levels, and vascular risks. Finally, we investigated whether regional neuronal injury, cerebral amyloidosis or vascular risks were independently associated with a diagnosis of LLOD. Because a diagnosis of MCI is considered to be associated with increased cerebral amyloidosis (Wolk et al., 2009), the vascular process (Casado Naranjo et al., 2015), and cortical atrophy (Jack et al., 2010), we also explored whether the association of LLOD with cerebral amyloidosis, vascular process, or regional cortical atrophy differs according to the presence of MCI.

## Materials and methods

### Participants

Elderly individuals who first experienced a major depressive disorder (MDD), as defined by the Diagnostic and Statistical Manual of Mental Disorders, 4th Edition (DSM-IV; First et al., 1996), after the age of 60 years were recruited from the Geriatric Psychiatry Clinic of the Seoul National University Hospital (SNUH) as LLOD subjects. In addition, elderly individuals without lifetime experience of MDD were recruited from the community as NC. To diagnose and characterize onset and course of MDD, as well as to exclude other psychiatric disorders, all participants underwent a standardized interview including the Structured Clinical Interview for DSM-IV (SCID) that is required for the diagnosis of MDD by board-certified psychiatrists at the time of recruitment. Reliable informants were also interviewed and medical records were reviewed for more accurate information. Exclusion criteria for both groups were: (1) dementia according to DSM-IV criteria; (2) comorbid major neuropsychiatric illnesses other than MDD; (3) a history of significant systemic illness/neurologic disorder; (4) major abnormal findings on brain MRI; (5) a history of electroconvulsive therapy; or, (6) contraindications for the MRI scan. The presence of MCI was not included in the exclusion criteria for both groups. Among the initially recruited 59 subjects, 3 LLOD participants were excluded from the final analysis due to significant abnormalities on MRI (i.e., acute infarct, old hemorrhage, and severe brain parenchymal tissue loss). The study was approved by the Institutional Review Board of SNUH and all participants provided written informed consent. This study was conducted in accordance with the recommendations of the current version of the Declaration of Helsinki.

### Clinical and neuropsychological assessment

Although all LLOD subjects were in MDD state at the time of enrollment, most of them (90%) were remitted from MDD after receiving treatment in the clinic at the time of the detailed examination including neuropsychological tests and MRI/PET scan. Severity of depressive symptoms at the time of the examination was measured using the 17-item Hamilton Rating Scale for Depression (Mulsant et al., 1994), the 30-item Korean version of the Geriatric Depression Scale (GDS; Yesavage et al., 1982; Bae and Cho, 2004) and Montgomery-Åsberg Depression Rating Scale (Montgomery and Asberg, 1979). All subjects received standardized clinical and neuropsychological assessments according to the protocol of the Korean version of the Consortium to Establish a Registry for Alzheimer Disease (CERAD) Assessment Packet (Morris et al., 1989; Lee et al., 2002), the CERAD neuropsychological battery (Lee et al., 2004) and the Stroop test (Seo et al., 2008) by board-certified psychiatrists and neuropsychologists. MCI was diagnosed according to the international consensus criteria (Winblad et al., 2004) and detailed information on the diagnostic criteria for MCI used in this study is described in elsewhere (see Supplementary Text).

The presence or absence of six VRFs, including hypertension, diabetes, dyslipidemia, heart disease, transient ischemic attack, and stroke was systematically assessed for each participant; the participant's history was provided by an informant and the medical records were reviewed. As a composite score of VRFs, the VRF score (VRS) was calculated as the number of the VRFs present and reported as a percentage (DeCarli et al., 2004). The systolic/diastolic blood pressure (SBP/DBP) was measured at supine position by trained nurse using sphygmomanometer and body mass index (BMI) were also assessed.

### MRI acquisition, pre-processing, and analyses

MRI scanning was performed using a 3T Siemens TrioTim magnetic resonance scanner (Siemens AG, Erlangen, Germany) to acquire three-dimensional (3-D) T1-weighted magnetization-prepared rapid gradient-echo (MPRAGE) and fluid-attenuated inversion recovery (FLAIR) sequences. Detailed information on MRI sequences and parameters is described in elsewhere (see Supplementary Text).

Voxel-based morphometry (VBM) analysis was performed using Statistical Parametric Mapping 8 (SPM8, Wellcome Department of Cognitive Neurology, London, UK; http://www.fil.ion.ucl.ac.uk/spm/) with the VBM8 toolbox (http://dbm.neuro.uni-jena.de/vbm/) to demonstrate group differences in regional gray matter (GM) density between the NC and LLOD groups. All T1-weighted images of each subject were normalized into standard anatomical space using MNI 152 template with a linear 12-parameter affine transformation. Next, normalized images were segmented into GM, white matter and cerebrospinal fluid. Smoothing at 12-mm full width at half maximum was performed after segmentation and modulation.

For the rating of white matter hyperintensity (WMH), all MRIs with FLAIR sequence were assessed blinded to clinical information by one experienced rater according to the Fazekas scale (Fazekas et al., 1987), where the severity of periventricular and deep WMH was scaled on a 0 (absence) to 3 (severe) separately.

### ^11^C-PiB PET image acquisition, pre-processing, and analyses

Participants also underwent ^11^C-PiB PET imaging using Biograph PET/CT scanners (Siemens, TN, USA). All participants underwent ^11^C-PiB PET imaging using Biograph PET/CT scanners (Siemens, TN, USA). For each subject, 550–750 MBq of ^11^C-PiB was administered by intravenous injection. A 20 min emission scan was obtained in 3D mode starting 50 min after injection and a CT scan was performed for attenuation correction (120 kVp, 40 mAs, pitches of 0.8). PET reconstructions were performed using a point spread function-based iterative algorithm (TrueX; 6 iterations, 21 subsets) with a matrix 256 × 256 in size (74 slices, voxel size: 1.3364 × 1.3364 mm^2^; slice thickness: 3 mm), and reconstructed images were rearranged onto transaxial, sagittal and coronal images.

Image preprocessing for statistical analyses was performed using SPM8 implemented in Matlab (MathWorks, Natick, MA, USA). The ^11^C-PiB PET data of each subject were co-registered to individual volumetric magnetic resonance images and then automatically spatially normalized into the standard MNI template in SPM8 using transformation parameters derived from the normalization of individual MRI scans to the template. All normalized images were reformatted with a 2 × 2 × 2 mm voxel. For quantitative normalization of cerebral ^11^C-PiB uptake values, the cerebellum was used as a reference region (Lopresti et al., 2005) and ^11^C-PiB retention maps, as region-to-cerebellar ratios, were generated by dividing regional uptake values by the individual mean cerebellar uptake values in the same images.

The automatic anatomic labeling algorithm (Tzourio-Mazoyer et al., 2002) and a region combining method (Reiman et al., 2009) were applied to set regions-of-interest (ROIs) to characterize ^11^C-PiB retention level in frontal, lateral parietal, posterior cingulate-precuneus (PC-PRC), lateral temporal and basal ganglia (BG) regions. Mean cortical amyloid burden was calculated for a global PiB retention index by averaging the mean ROI value except BG. The image was classified as PiB-positive if the standardized uptake value ratio (SUVR) of ^11^C-PiB retention was 1.4 or higher in one of the following cortical ROIs: frontal, lateral temporal, lateral parietal, or PC-PRC (Choe et al., 2014).

### Plasma Aβ peptide level assessment and apolipoprotein E (*APOE*) genotyping

Fasting blood samples were collected in the morning by venipuncture in tubes containing EDTA as anticoagulant. After centrifugation, plasma samples were aliquoted into polypropylene tubes and stored at −80°C pending biochemical analyses without being thawed and refrozen. Quantification of plasma Aβ isoforms was performed using INNO-BIA plasma Aβ forms assays (Innogenetics, Ghent, Belgium) and the Bio-Plex 200 system with high throughput fluidics (BIO-RAD, Hercules, CA, USA) based on a previous study (Hansson et al., 2010). Genomic DNA was extracted from venous blood and *APOE* genotyping was performed according to the method described previously (Wenham et al., 1991).

### Statistical analyses

For comparison of demographic and clinical variables between the two groups, independent *t*-test for continuous variables and chi-square or Fisher's exact test for categorical variables was used (*p* < 0.05, two-sided). Neuropsychological variables were compared between the two groups using analysis of covariance (ANCOVA) with age, gender, educational level and GDS score as covariates. VBM analysis was performed to identify the region where LLOD subjects showed greater cortical atrophy compared with NC at both uncorrected *p* < 0.001 (*k* = 100) and family-wise error (FWE)-corrected *p* < 0.05 (*k* = 100), after controlling for age, gender and educational level. Comparison of PiB retention level, as well as plasma Aβ peptide level, between the two groups was performed using ANCOVA after controlling for age, gender and educational level. Multiple logistic regression analysis with diagnostic state (NC vs. LLOD) as a dependent variable was performed to investigate the independent association of regional neuronal injury, cerebral amyloidosis, and vascular risk with occurrence of LLOD after controlling for age, gender, and educational level. All of the abovementioned analyses were performed again after excluding LLOD subjects with MCI (LLOD_MCI_) to characterize the LLOD subjects without MCI (LLOD_woMCI_) and vice versa.

## Results

### Demographic, clinical, and neuropsychological characteristics

Differences in terms of age, gender, and educational level, as well as *APOE* ε4 carrier frequency, were not significant between LLOD subjects and NC (Table 1). Although 26 out of 29 LLOD patients were remitted from MDD at the time of examination, LLOD patients had significantly higher scores on the depression symptom scale than NC. In addition, 48% of LLOD subjects and none of the NC were diagnosed with comorbid MCI.

**Table 1:** **Demographic and clinical characteristics of the subjects**.

	**NC (*N* = 27)**	**LLOD (*N* = 29)**	**LLOD_woMCI_ (*N* = 15)**	**LLOD_MCI_ (*N* = 14)**
**DEMOGRAHPIC CHARACTERISTICS**				
Age (years)	68.70 ± 6.06	71.62 ± 5.08	71.40 ± 5.11	71.86 ± 5.23
Gender (M/F)	14/13	8/21	6/9	2/12^*^
Educational level (years)	10.81 ± 4.52	8.69 ± 4.54	9.93 ± 4.94	7.36 ± 3.80^*^
*APOE* ε4 carrier/non-carrier	5/22	3/26	0/15	3/11
**CLINICAL CHARACTERISTICS**				
Age at onset of first MDD (years)	–	68.12 ± 4.49	67.27 ± 4.47	69.04 ± 4.50
Duration since first MDD onset (years)	–	3.50 ± 3.13	4.13 ± 3.44	2.82 ± 2.71
GDS	5.85 ± 3.96	12.00 ± 5.76^**^	12.00 ± 5.96^**^	12.00 ± 5.75^**^
HRSD	1.22 ± 1.67	4.66 ± 3.95^**^	4.61 ± 3.20^*^	4.71 ± 4.75^*^
MADRS	1.70 ± 2.05	6.79 ± 5.57^**^	5.87 ± 2.97^**^	8.14 ± 7.38^*^
Subtype of MCI (aMCI/naMCI)	–	–	–	6/8
**VASCULAR RISKS EVALUATION**				
VRS (%)	19.75 (15.36)	16.67 (14.77)	17.78 (14.73)	15.48 (15.28)
BMI (kg/m^2^)	24.85 ± 2.92	23.45 ± 3.16	23.34 ± 3.72	23.57 ± 2.57
SBP (mmHg)	118.81 ± 9.82	126.52 ± 13.23^*^	127.60 ± 14.75^*^	125.36 ± 11.82^a^
DBP (mmHg)	79.56 ± 7.53	78.14 ± 8.56	78.73 ± 8.98	77.50 ± 8.37
Use of antihypertensives, *N* (%)	15 (55.6)	13 (44.8)	8 (53.3)	5 (35.7)
SBP_under antihypertensives^b^	119.20 ± 10.47	128.23 ± 10.67^*^	129.63 ± 13.21	126.00 ± 5.15
DBP_under antihypertensives^b^	78.13 ± 8.50	77.31 ± 7.32	79.25 ± 7.17	74.20 ± 7.16
Periventricular WMH	0.96 ± 0.76	1.31 ± 0.60^c^	1.27 ± 0.59	1.36 ± 0.63
Deep WMH	1.26 ± 0.45	1.31 ± 0.47	1.33 ± 0.49	1.29 ± 0.47

*Data are presented as mean ± SD or numbers*.

* *Significant difference compared to NC (p < 0.05)*.

** *Significant difference compared to NC (p < 0.001)*.

a *SBP of LLOD_MCI_ was higher than NC but did not reach statistical significance (p = 0.067)*.

b *Data are presented as mean ± SD among subjects who took antihypertensives*.

c *Mean of periventricular WMH severity was higher in LLOD group compared to NC; but, it was not statistically significant (p = 0.062)*.

*NC, Normal Controls; LLOD, Late-life Onset Depression; MCI, Mild Cognitive Impairment; LLOD_woMCI_, LLOD without MCI; LLOD_MCI_, LLOD with MCI; APOE, Apolipoprotein E; MDD, Major Depressive Disorder; GDS, The Korean version of the Geriatric Depression Scale; HRSD, Hamilton Rating Scale of Depression; MADRS, Montgomery-Åsberg Depression Rating Scale; aMCI, amnestic MCI; naMCI, non-amnestic MCI; VRS, Vascular Risk Factor Score; BMI, Body Mass Index; SBP, Systolic Blood Pressure; DBP, Diastolic Blood Pressure; WMH, White Matter Hyperintensity*.

In terms of vascular risk evaluation, no significant differences in VRS, BMI, and DBP were observed between NC and LLOD subjects. However, SBP was significantly higher in LLOD subjects than in NC. In subgroup analysis, LLOD_woMCI_ subjects had significantly higher SBP than NC. Mean of SBP in LLOD_MCI_ subjects was higher than that of NC group; but, it was not statistically significant. Even among the subjects treated with antihypertensives, SBP of LLOD group was still significantly higher than NC. Additionally, mean of periventricular WMH severity was higher in LLOD group compared to NC; but, it was not statistically significant.

Compared with NC, LLOD subjects showed significantly greater impairments on multiple cognitive tests including the Mini-mental State Examination (MMSE), Semantic fluency, Word-list memory and Constructional praxis of the CERAD neuropsychological battery, and all Stroop tests (Supplementary Table 1).

### Regional cortical atrophy in LLOD

**Figure 1:**
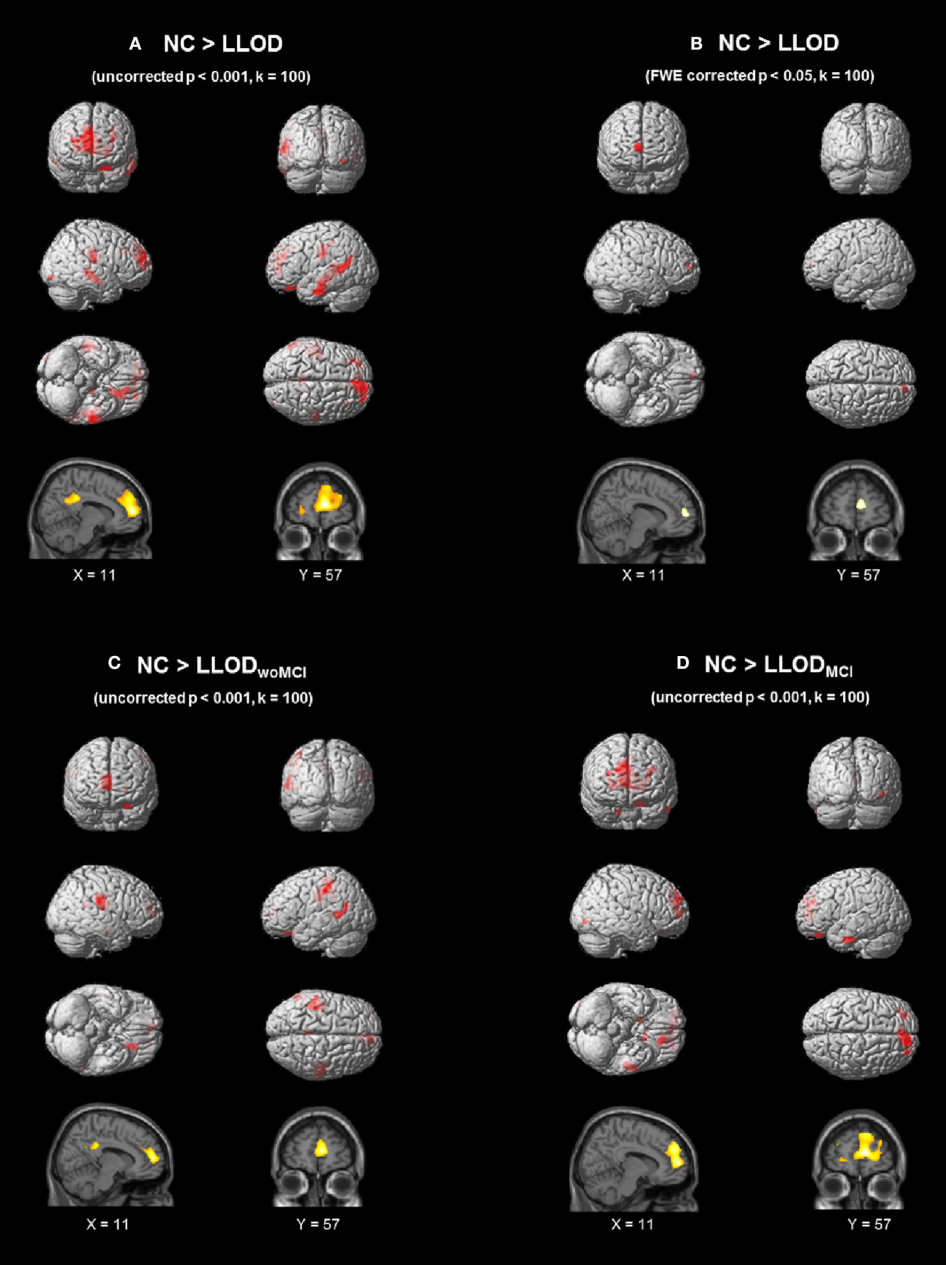
**Regional GM atrophy of LLOD subjects compared to NC after controlling age, gender, and educational level (A) at uncorrected ***p*** < 0.001 (***k*** = 100) and (B) FWE-corrected ***p*** < 0.05 (***k*** = 100)**. **(C)** and **(D)** demonstrated regional GM atrophy in subjects with **(C)** LLOD_woMCI_ and **(D)** LLOD_MCI_ at uncorrected *p* < 0.001 (*k* = 100). NC, Normal Controls; LLOD, Late-life Onset Depression; MCI, Mild Cognitive Impairment; LLOD_woMCI,_ LLOD without MCI; LLOD_MCI,_ LLOD with MCI; GM, Gray Matter; FWE, Family-wise Error.

On VBM analysis, LLOD individuals demonstrated regional cortical atrophy in the bilateral prefrontal regions including the medial prefrontal areas as well as the bilateral parietal and temporal regions compared with NC at uncorrected *p* < 0.001, *k* = 100 (Figure 1A and Supplementary Table 2). After correction for multiple comparisons (FWE-corrected *p* < 0.05, *k* = 100), LLOD subjects still showed significant prefrontal atrophy mainly in the right medial prefrontal region (Figure 1B). Prefrontal cortical atrophy with peak voxel located in the right medial prefrontal region (peak MNI: 11, 57, 9) was similarly observed when LLOD_woMCI_ and LLOD_MCI_ subjects were compared to NC separately at uncorrected *p* < 0.001, *k* = 100 (Figures 1C,D and Supplementary Table 3).

### Cerebral PiB retention and plasma Aβ peptide level in LLOD

**Figure 2:**
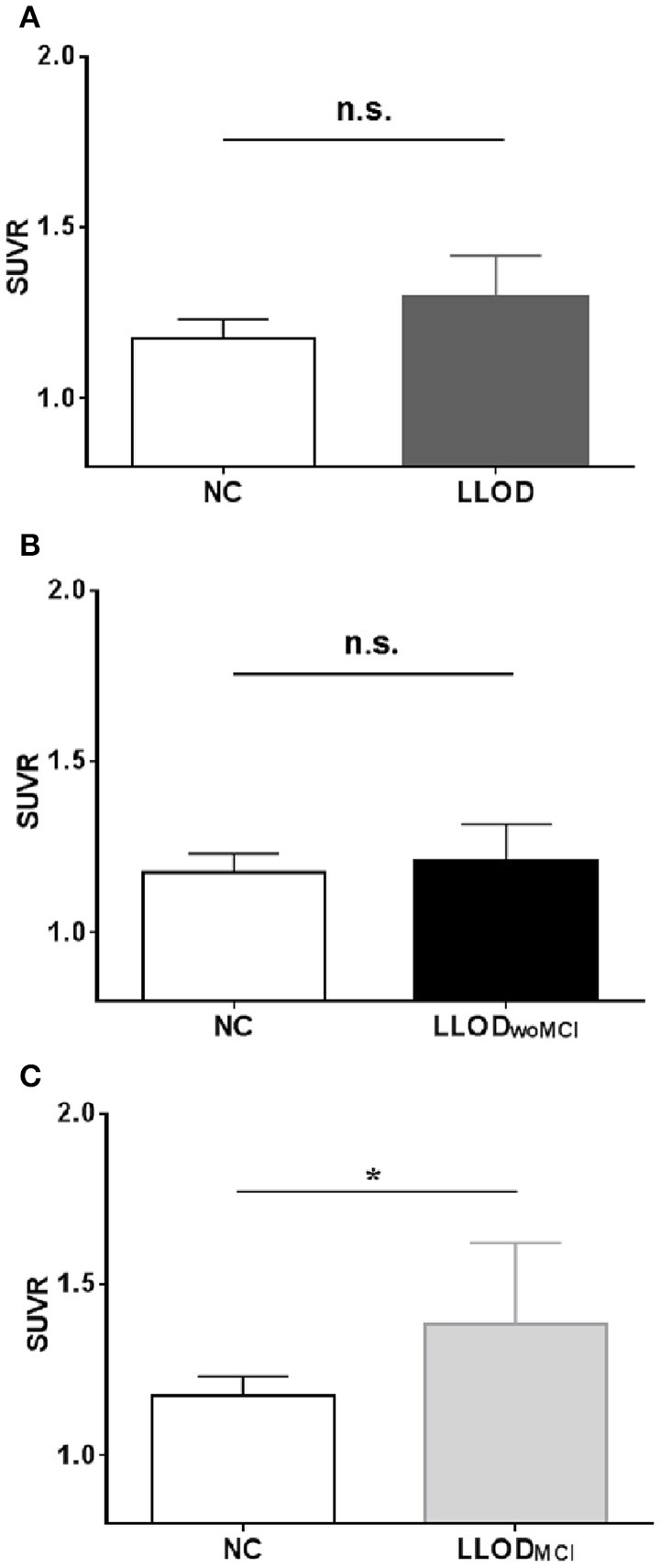
**Mean cortical PiB retention level (SUVR) in subjects: (A) NC vs. LLOD, (B) NC vs. LLOD_**woMCI,**_(C) NC vs. LLOD_**MCI.**_** ^*^p < 0.05 (after controlling the effect of age, gender, and educational level). PiB, Pittsburgh Compound B; NC, Normal Controls; LLOD, Late-life Onset Depression; MCI, Mild Cognitive Impairment; LLOD_woMCI,_ LLOD without MCI; LLOD_MCI_, LLOD with MCI; SUVR, Standardized Uptake Value Ratio.

Overall, LLOD patients showed no significant differences in both global PiB retention and the PiB-positivity rate compared with NC (Table 2 and Figure 2). Regional PiB retention in the frontal, lateral parietal, PC-PRC, lateral temporal, or BG regions was also not significantly different between NC and LLOD subjects. In terms of plasma Aβ peptides level, no significant differences were observed in all plasma Aβ peptide measurements or their ratios between NC and LLOD subjects (Table 3).

**Table 2:** **Cerebral PiB retention level of subjects**.

	**NC (*N* = 27)**	**LLOD (*N* = 29)**	**LLOD_woMCI_ (*N* = 15)**	**LLOD_MCI_ (*N* = 14)**
**REGIONAL PiB RETENTION**				
Frontal region	1.20 ± 0.15	1.31 ± 0.34	1.21 ± 0.18	1.42 ± 0.44^*^^a^
Lateral parietal region	1.08 ± 0.15	1.20 ± 0.32	1.14 ± 0.26	1.27 ± 0.38^*^^a^
PC-PRC region	1.23 ± 0.18	1.36 ± 0.36	1.28 ± 0.29	1.45 ± 0.42^*^^a^
Lateral temporal region	1.15 ± 0.11	1.26 ± 0.30	1.21 ± 0.17	1.33 ± 0.39^b^
BG	1.32 ± 0.14	1.38 ± 0.24	1.35 ± 0.15	1.43 ± 0.31
**GLOBAL PiB RETENTION**				
Mean cortical PiB retention	1.18 ± 0.14	1.29 ± 0.32	1.21 ± 0.19	1.38 ± 0.41^*^^a^
PiB-positivity (%)	3 (11.1)	8 (27.6)	2 (13.3)	6 (42.9)^*^^c^

*Data are presented as mean ± SD or number (%). Regional and global PiB retention levels are shown as a standardized uptake value ratio (SUVR). For comparison of continuous variables, analysis of covariance test was used with age, gender, and educational level as covariates and chi-square test or Fisher's exact test was used for the comparison of categorical variable*.

* *Significant difference compared to NC (p < 0.05)*.

a *LLOD_MCI_ subjects showed significantly higher PiB SUVR in frontal (p = 0.040), lateral parietal (p = 0.027), PC-PRC (p = 0.031) and mean cortical (p = 0.036) regions compared to NC after controlling the effect of age, gender, and educational level*.

b *LLOD_MCI_ subjects had higher PiB SUVR in the lateral temporal region at marginal significance after controlling the effect of age, gender, and educational level (p = 0.051)*.

c *PiB-positivity rate was significantly higher in LLOD_MCI_ subjects compared to NC (p = 0.042, Fisher's exact test)*.

*PiB, Pittsburgh Compound B; NC, Normal Controls; LLOD, Late-life Onset Depression; MCI, Mild Cognitive Impairment; LLOD_woMCI,_ LLOD without MCI; LLOD_MCI,_ LLOD with MCI; PC-PRC, Posterior Cingulate-Precuneus; BG, Basal Ganglia; SUVR, Standardized Uptake Value Ratio*.

**Table 3:** **Plasma Aβ peptides level of subjects**.

	**NC (*N* = 27)**	**LLOD (*N* = 29)**	**LLOD_woMCI_ (*N* = 15)**	**LLOD_MCI_ (*N* = 14)**
Plasma Aβ_1–42_	39.58 ± 7.82	43.51 ± 11.19	43.22 ± 10.71	43.81 ± 12.08^*^^a^
Plasma Aβ_1–40_	176.03 ± 17.83	189.74 ± 35.68	179.54 ± 35.05	200.67 ± 34.21^*^^a^
Plasma Aβ_*N–*42_	27.95 ± 6.05	30.56 ± 9.29	30.86 ± 8.49	30.24 ± 10.40
Plasma Aβ_*N–*40_	186.81 ± 22.54	195.19 ± 39.34	200.85 ± 45.49	189.12 ± 32.08
Aβ_1–40_/Aβ_1–42_	4.61 ± 1.03	4.60 ± 1.32	4.35 ± 1.14	4.87 ± 1.47
Aβ_*N–*40_/Aβ_*N–*42_	7.05 ± 2.13	7.13 ± 3.44	7.07 ± 2.82	7.21 ± 4.12

*Data are presented as mean ± SD. Plasma Aβ peptide levels are shown as ng/L*.

* *Significant difference compared to NC (p < 0.05)*.

a *LLOD_MCI_ subjects had significantly higher plasma Aβ_1–42_ and plasma Aβ_1–40_ peptides level compared to NC (p = 0.03 and p = 0.006, respectively) after controlling the effect of age, gender and educational level*.

*NC, Normal Controls; LLOD, Late-life Onset Depression; MCI, Mild Cognitive Impairment; LLOD_woMCI,_ LLOD without MCI; Aβ, Beta-amyloid*.

In subgroup analysis, LLOD_woMCI_ subjects did not show significant differences in global or regional PiB retention levels, or in the PiB-positivity rate compared with NC. Similarly, plasma Aβ peptides levels were not different between LLOD_woMCI_ subjects and NC. However, LLOD_MCI_ subjects had significantly more PiB-positive cases and showed higher global and regional PiB retention than NC (Table 2 and Figure 2). In addition, plasma Aβ_1–42_ and Aβ_1–40_ peptide levels were significantly higher in LLOD_MCI_ subjects than NC (Table 3).

### Independent association of regional neuronal injury, cerebral amyloidosis, and vascular process with LLOD diagnosis

Multiple logistic regression analysis with diagnostic state (NC vs. LLOD) as a dependent variable was performed to investigate the independent relationship of regional neuronal injury, cerebral amyloidosis, and vascular risk with LLOD diagnosis. For this analysis, regional neuronal injury was defined as mean GM density extracted from a prefrontal ROI that was set as a cluster where LLOD subjects showed reduced GM density at FWE-corrected *p* < 0.05 (peak MNI: 11, 57, 9; *T* = 5.79; 463 voxels). In addition to prefrontal ROI GM density, global PiB retention, as a measure of cerebral amyloidosis, and SBP, a vascular risk that was significantly different between NC and LLOD subjects, were included in the regression model as independent variables. After adjusting for age, gender and educational level, only prefrontal ROI GM density showed a significant association with LLOD diagnosis [odds ratio (OR) = 0.47 per 0.01; 95% confidence interval (CI): 0.29 – 0.75], whereas global PiB retention (OR = 1.07 per 0.01 SUVR; 95% CI: 0.99 – 1.15) and SBP (OR = 1.06 per 1 mmHg; 95% CI: 0.99 – 1.14) did not in the same model. Moreover, prefrontal ROI GM density was significantly associated with LLOD_woMCI_ diagnosis (OR = 0.25 per 0.01; 95% CI 0.07 – 0.85) or LLOD_MCI_ diagnosis (OR = 0.56 per 0.01; 95% CI 0.36 – 0.86) in the regression models, but global PiB retention and SBP were not.

## Discussion

We found that nearly half of the subjects who experienced LLOD had comorbid cognitive impairment compatible with MCI, which was not observed in NC. LLOD, irrespective of comorbid MCI diagnosis, was associated with prominent prefrontal cortical atrophy. The LLOD_MCI_ subgroup showed increased cerebral PiB retention and plasma Aβ peptide levels as measures of cerebral amyloidosis, compared with NC, while overall LLOD and LLOD_woMCI_ subjects did not. In terms of the vascular process, LLOD individuals had higher SBP than NC, particularly when MCI did not coexist. When simultaneously analyzed in the same model, only prefrontal cortical atrophy showed a significant independent association with LLOD diagnosis, irrespective of MCI status, while cerebral PiB retention and SBP did not. To the best of our knowledge, this study is the first to investigate both *in vivo* cerebral amyloidosis and regional cortical atrophy simultaneously with vascular risks in the context of LLOD occurrence.

As the accumulation of brain Aβ protein begins 15–20 years before the onset of dementia in AD (Sperling et al., 2011), we aimed to investigate whether LLOD is a secondary result of brain Aβ deposition in the prodromal period of AD dementia. Thus, we strictly recruited only elderly subjects with late-life onset major depression, excluding ELOD cases to increase the homogeneity of study participants. Reportedly, ELOD is a risk factor for developing AD dementia rather than a prodromal phenomenon of AD dementia (Ownby et al., 2006; Byers and Yaffe, 2011). Recent reports on changes in the serum or plasma Aβ_1–40/_Aβ_1–42_ ratio even in young-aged major depression patients (Baba et al., 2012) or elderly subjects with ELOD (Pomara et al., 2006; Namekawa et al., 2013) support the link between ELOD and altered Aβ metabolism. Preclinical evidence also suggests that stress responses related to depressive experiences contribute to Aβ accumulation by initiating increased Aβ production (Green et al., 2006; Kang et al., 2007) or interacting with the amyloidogenic process linked to the serotonergic system (Sierksma et al., 2010). Several previous studies have reported elevated brain Aβ deposition or altered plasma Aβ biomarkers in elderly depression (Qiu et al., 2007; Sun et al., 2008; Wu et al., 2014). However, because these studies did not strictly define elderly depression as LLOD, the findings were likely influenced by an etiological contribution of earlier-onset depression to the Aβ-related pathology such that comparison with our results is difficult.

Although most LLOD subjects were in the remitted state, approximately half had comorbid MCI, which was not observed in NC. Considering that structural and functional brain impairments are already prominent in the MCI state (Pihlajamaki et al., 2009; Jack et al., 2010), the higher frequency of comorbid MCI in LLOD subjects indicates that brain damage or pathology is potentially more prevalent in this group, as reported previously (Weisenbach and Kumar, 2014). Our study also revealed prominent prefrontal cortical atrophy in LLOD subjects, regardless of MCI diagnosis. The prefrontal region, especially the medial prefrontal cortex, is important in emotional processing (Elliott and Dolan, 2003). Structural abnormality in the prefrontal region is a robust finding in previous structural imaging studies on late-onset major depression (Kumar et al., 1998) as well as overall MDD (Steele et al., 2007; Lorenzetti et al., 2009). Reductions in neuronal cell size and density in the prefrontal region in MDD were consistently found in previous studies (Kim et al., 2016). Thus, our findings from structural MRI analysis of the cerebral cortex, together with other prior evidences, support that prefrontal neuronal injury is likely a common neural substrate for the emergence of LLOD as well as MDD in general, although underlying etiological factors could be heterogeneous (Butters et al., 2008b; Weisenbach and Kumar, 2014).

While the regional neuronal injury was closely associated with LLOD and did not differ according to the comorbid MCI state, the relationship between cerebral amyloidosis and LLOD depended on the presence of MCI. LLOD_MCI_ was closely related with increased cerebral PiB retention and plasma Aβ_1–42_ and Aβ_1–40_ peptide levels, whereas overall LLOD and LLOD_woMCI_ were not. These findings suggest that cerebral amyloidosis is not the major determinant of LLOD in elderly individuals without cognitive impairment compatible with MCI, but probably exerts an influence on the occurrence of LLOD in subjects who are in the MCI state. Our findings on the differential association of cerebral amyloidosis and LLOD according to MCI comorbidity might also explain inconsistencies among previous findings: elderly subjects with current or history of depression showed increased brain Aβ level when subjects had comorbid MCI diagnosis, whereas those without MCI did not compared to NC (Butters et al., 2008a; Wu et al., 2016). In addition, the proportion of MCI comorbidity in study subjects as well as methodological differences in plasma Aβ measurements might contribute to the mixed results of previous studies on plasma Aβ level in elderly individuals with depression (Pomara et al., 2006; Qiu et al., 2007; Sun et al., 2008; Song et al., 2011; Baba et al., 2012; Namekawa et al., 2013; Osorio et al., 2014).

We also found that SBP in LLOD patients was significantly higher than in NC, particularly in LLOD_woMCI_ subjects. LLOD also tended to have higher WMH than NC. These results support the previous vascular depression hypothesis (Alexopoulos et al., 1997a) and indicate that the vascular process may have a greater impact on the occurrence of LLOD in subjects who are not in the MCI state and have a low probability of underlying AD processes such as cerebral amyloidosis. Progressive cortical thinning in the prefrontal region has also been reported in hypertensive individuals (Gonzalez et al., 2015), suggesting that the prefrontal cortical region is selectively susceptible to blood pressure elevation.

When analyzed in the same multiple logistic regression model that included prefrontal GM density, cerebral amyloidosis and SBP as independent variables, only prefrontal GM density showed a significant independent association with LLOD diagnosis, regardless of MCI comorbidity state, which was not observed for global PiB retention or SBP. These results indicate that cerebral amyloidosis and elevated SBP only indirectly contribute to LLOD occurrence *via* prefrontal neuronal injury.

The present study had several limitations. First, it used a cross-sectional design and was limited in terms of providing information on the causal contribution of cerebral amyloidosis or blood pressure to LLOD. Second, the sample size for each LLOD subgroup analysis, although conducted for exploratory purposes, was relatively small, which might have reduced the statistical power. Further studies with larger sample sizes and longitudinal follow-ups are needed to address these issues.

In conclusion, our findings suggest that prefrontal neuronal injury is probably a common brain alteration underlying the occurrence of LLOD. Additionally, cerebral amyloidosis might be associated with LLOD via prefrontal neuronal injury in the MCI stage, in which structural and functional brain impairment caused by the AD process begins to become prominent. Meanwhile, the vascular process, SBP elevation in particular, is associated with the occurrence of LLOD through prefrontal neuronal injury even in cognitively intact or less impaired individuals. From a clinical standpoint, an LLOD patient likely has underlying AD pathologies in his/her brain, if the patient is still in the MCI state even after the remission of depression. However, this possibility is low if the patient is cognitively less impaired and not in the MCI state after the remission of depression. Instead, we should consider that vascular process is a main contributor to LLOD in such cases.

## Author contributions

MB and DL contributed to the design of the study, data collection, data analyses, data interpretation, and prepared the report. YC, BS, JH, HC, HB, JL, HK, JW were involved in the collection and analyses of clinical data as well as drafting of the report. DY, JP, YK, EY, and CS participated in image data preprocessing, analyses, and were involved in preparation of the report.

### Conflict of interest statement

The authors declare that the research was conducted in the absence of any commercial or financial relationships that could be construed as a potential conflict of interest.
