# Supplementary material for: Association of Cerebral Amyloidosis, Blood Pressure, and Neuronal Injury with Late-Life Onset Depression
Source: Front Aging Neurosci. 2016 Oct 13;8:236. doi: 10.3389/fnagi.2016.00236 (PMC5061734; doi:10.3389/fnagi.2016.00236)
Supplement: Supplementary file 1 [file Table1.PDF]

Supplementary Table 1. Neuropsychological characteristics of the subjects.

	NC (N = 27)	LLOD (N = 29)	$LLOD_{woMCI}$ (N = 15)	$LLOD_{MCI}$ (N = 14)
Neuropsychological tests (1) : Raw score				
MMSE	28.04 ± 1.81	25.28 ± 2.39**	26.53 ± 1.92	23.93 ± 2.13**
Semantic fluency	16.00 ± 4.17	12.17 ± 2.39*	12.80 ± 2.31	11.50 ± 2.38
Boston naming test	12.19 ± 1.71	11.55 ± 1.74	12.07 ± 2.05	11.00 ± 1.18
Word-list immediate memory	17.56 ± 2.78	14.72 ± 2.60*	15.40 ± 2.38	14.0 ± 2.72*
Word-list recall	6.04 ± 1.32	4.86 ± 1.66	5.13 ± 1.68	4.57 ± 1.65*
Word-list recognition	9.33 ± 0.96	8.83 ± 1.79	9.00 ± 2.10	8.64 ± 1.45
Constructional praxis	10.67 ± 0.88	8.83 ± 2.02**	9.47 ± 2.29*	8.14 ± 1.46**
Constructional praxis recall	7.67 ± 2.87	4.97 ± 3.31	6.07 ± 3.84	3.79 ± 2.19*
Stroop word	67.19 ± 11.33	51.59 ± 13.29*	53.53 ± 16.47*	49.50 ± 8.90*
Stroop color	57.52 ± 10.35	44.14 ± 14.20*	47.87 ± 17.20	40.14 ± 9.09*
Stroop color-word	43.78 ± 9.86	29.48 ± 9.11**	32.80 ± 10.60*	25.93 ± 5.61**
Neuropsychological tests (2) : Age-, gender- and education-adjusted z-score				
MMSE	0.33 ± 0.86	-0.76 ± 1.05	-0.37 ± 0.97	-1.18 ± 1.00
Semantic fluency	0.29 ± 0.90	-0.55 ± 0.60	-0.44 ± 0.53	-0.67 ± 0.66
Boston naming	0.61 ± 0.56	0.50 ± 0.74	0.64 ± 0.80	0.35 ± 0.68
Word-list memory	0.27 ± 0.60	-0.49 ± 0.66	-0.32 ± 0.54	-0.67 ± 0.73
Word-list recall	0.07 ± 0.64	-0.47 ± 0.78	-0.33 ± 0.79	-0.63 ± 0.76
Word-list recognition	0.21 ± 0.73	-0.06 ± 1.07	0.09 ± 1.17	-0.22 ± 0.97
Constructional praxis	0.61 ± 0.40	-0.53 ± 1.27	-0.13 ± 1.21	-0.97 ± 1.22
Constructional praxis recall	0.30 ± 0.90	-0.48 ± 1.05	-0.20 ± 1.21	-0.78 ± 0.79
Stroop word	0.05 ± 0.76	-0.96 ± 1.04	-0.86 ± 1.19	-1.08 ± 0.89
Stroop color	0.18 ± 0.93	-1.11 ± 1.47	-0.73 ± 1.62	-1.52 ± 1.21
Stroop color-word	0.81 ± 0.86	-0.50 ± 0.90	-0.21 ± 0.98	-0.82 ± 0.72

Data are presented as mean ± SD.
*Significant difference compared to NC ($p < 0.05$)
**Significant difference compared to NC ($p < 0.001$)

NC, Normal Controls; LLOD, Late-life Onset Depression; MCI, Mild Cognitive Impairment; $LLOD_{woMCI}$, LLOD without MCI; $LLOD_{MCI}$, LLOD with MCI; MMSE, Mini-mental Status Examination
