# Supplementary material for: Association of Cerebral Amyloidosis, Blood Pressure, and Neuronal Injury with Late-Life Onset Depression
Source: Front Aging Neurosci. 2016 Oct 13;8:236. doi: 10.3389/fnagi.2016.00236 (PMC5061734; doi:10.3389/fnagi.2016.00236)
Supplement: Supplementary file 2 [file Table2.PDF]

Supplementary Table 2. Anatomical regions shown reduced regional GM density in LLOD subjects compared to NC (cluster size > 100 voxels)

Anatomical regions	Peak coordinates (MNI)			Side	Cluster size	T-value
	x	y	z			
FWE-corrected p < 0.05						
Medial frontal	11	57	9	R	463	5.79
Uncorrected p < 0.001						
Medial frontal	11	57	9	R	9487	5.79
Orbitofrontal	-21	33	-26	L	1464	4.54
Middle frontal	35	24	27	R	303	3.99
Posterior cingulate	9	-37	34	R	2729	4.48
	0	-65	11	L	243	3.82
Parietotemporal	50	-22	30	R	4077	4.63
Inferior parietal	-47	-19	30	L	2243	4.44
Lateral temporal	-56	-42	9	L	6365	4.16
Parahippocampal	-18	-18	-35	L	136	3.87
Lateral occipital	33	-84	-8	R	313	3.96
Pontine tegmentum	5	-36	-27	R	137	3.81

GM, Gray Matter; NC, Normal Controls; LLOD, Late-life Onset Depression; FWE-corrected, Family-wise Error-corrected; R, Right; L, Left.
