# Supplementary material for: Association of Cerebral Amyloidosis, Blood Pressure, and Neuronal Injury with Late-Life Onset Depression
Source: Front Aging Neurosci. 2016 Oct 13;8:236. doi: 10.3389/fnagi.2016.00236 (PMC5061734; doi:10.3389/fnagi.2016.00236)
Supplement: Supplementary file 3 [file Table3.PDF]

Supplementary Table 3. Anatomical regions shown reduced regional GM density in $LLOD_{woMCI}$ and $LLOD_{MCI}$ subjects compared to NC (uncorrected p < 0.001, cluster size > 100 voxels)

Anatomical regions	Peak coordinates (MNI)			Side	Cluster size	T-value
	**x**	**y**	**z**			
NC > $LLOD_{woMCI}$						
Medial frontal	8	56	7	R	1965	4.96
Orbitofrontal	-26	30	-26	L	564	4.24
Anterior cingulate	-20	51	3	L	195	3.97
Posterior cingulate	-6	-40	40	L	1580	4.29
Lateral parietal	-45	-37	54	L	2785	4.49
	48	-21	28	R	2260	5.43
Lateral temporal	-51	-51	4	L	1477	4.04
	42	-46	18	R	293	3.74
	51	-9	-18	R	104	3.52
NC > $LLOD_{MCI}$						
Medial Frontal	11	57	9	R	7689	5.42
	-14	9	-21	L	375	3.61
Orbitofrontal	-20	36	-27	L	615	4.68
Uncus	17	3	-35	R	199	3.99
Lateral occipital	42	-84	-6	R	232	3.89
Posterior cingulate	3	-64	13	R	218	3.87
Inferior temporal	-60	-6	-32	L	791	3.77

GM, Gray Matter; NC, Normal Controls; LLOD, Late-life Onset Depression; MCI, Mild Cognitive Impairment; $LLOD_{woMCI}$, LLOD without MCI; $LLOD_{MCI}$, LLOD with MCI; R, Right; L, Left.
