# Supplementary material for: Association of Cerebral Amyloidosis, Blood Pressure, and Neuronal Injury with Late-Life Onset Depression
Source: Front Aging Neurosci. 2016 Oct 13;8:236. doi: 10.3389/fnagi.2016.00236 (PMC5061734; doi:10.3389/fnagi.2016.00236)
Supplement: Supplementary file 4 [file Supplementary_Text.pdf]

Supplementary Material

Association of Cerebral Amyloidosis, Blood Pressure, and Neuronal Injury with Late-life Onset Depression

Min Soo Byun, Young Min Choe, Bo Kyung Sohn, Dahyun Yi, Ji Young Han, Jinsick Park, Hyo Jung Choi, Hyewon Baek, Jun Ho Lee, Hyun Jung Kim, Yu Kyeong Kim, Eun Jin Yoon, Chul-Ho Sohn, Jong Inn Woo, Dong Young Lee*

*** Correspondence:** Dong Young Lee: selfpsy@snu.ac.kr

1 Supplementary Text

1.1 Diagnostic criteria for mild cognitive impairment (MCI)

The diagnostic criteria for MCI based on the international consensus criteria (Winblad et al., 2004) are as follows: 1) neither normal nor demented, 2) presence of self and/or informant report of cognitive decline, 3) impairment on objective cognitive tasks, and 4) independence in functional activities. The global clinical dementia rating was 0.5 for all MCI participants. As for the criterion 3, the impairment of objective cognitive task was defined if the age-, gender- and education-adjusted z-score of neuropsychological tests was ≤ -1.5. Amnestic MCI was diagnosed if z-score of one or more memory domain tests (i.e. Word memory list, Word memory recall, Word memory recognition and Constructional praxis recall of the CERAD neuropsychological battery) was ≤ -1.5. Non-amnestic MCI was diagnosed if z-score of at least one of the neuropsychological tests other than memory (i.e. Semantic fluency, Boston naming test and Constructional praxis of the CERAD neuropsychological battery, and all Stroop tests) was ≤ -1.5.

1.2 MRI acquisition: sequences and parameters

MRI scanning was performed using a 3T Siemens TrioTim magnetic resonance scanner (Siemens AG, Erlangen, Germany) to acquire three-dimensional (3-D) T1-weighted magnetisation-prepared rapid gradient-echo (MPRAGE) and fluid-attenuated inversion recovery (FLAIR) sequences. 3-D T1- MPRAGE sequence images were obtained using the following parameters: repetition time (TR) = 1900 ms; echo time (TE) = 3.1 ms; field of view (FOV) = 240 × 240 mm^2; flip angle (FA) = 9°; slice thickness = 1 mm. In addition, following parameters were used for FLAIR sequence: TR = 5000 ms; TE = 348 ms; FOV = 250 × 250 mm^2; FA = 120°; slice thickness = 1mm.

References

Winblad, B., Palmer, K., Kivipelto, M., Jelic, V., Fratiglioni, L., Wahlund, L.O., et al. (2004). Mild cognitive impairment--beyond controversies, towards a consensus: report of the International Working Group on Mild Cognitive Impairment. *J Intern Med* 256(3), 240-246. doi: 10.1111/j.1365-2796.2004.01380.x.
